# Tumor-secreted factors induce IL-1β maturation via the glucose-mediated synergistic axis of mTOR and NF-κB pathways in mouse macrophages

**DOI:** 10.1371/journal.pone.0209653

**Published:** 2018-12-26

**Authors:** Yunseo Woo, Hyeran Kim, Keun-Cheol Kim, Jeong A. Han, Yu-Jin Jung

**Affiliations:** 1 Department of Biological Sciences, Kangwon National University, Chuncheon, Gangwon, Republic of Korea; 2 Department of Biochemistry and Molecular Biology, School of Medicine, Kangwon National University, Chuncheon, Gangwon, Republic of Korea; Indian Institute of Science Education and Research, INDIA

## Abstract

Macrophages are one of the major cell types that produce IL-1β. IL-1β maturation occurs via inflammasome activation, and mature IL-1β is then released from the cell. Secreted IL-1β mediates inflammatory reactions in various pathological environments, such as those in infectious, autoimmune, and cancerous diseases. Although the mechanism of IL-1β production has been discovered in infectious and autoimmune diseases, its production mechanism in the tumor microenvironment is unclear. Therefore, the mechanism of IL-1β production in macrophages in the tumor microenvironment was investigated in this study. First, bone marrow-derived macrophages obtained from C57BL/6 mice were treated with B16F10 tumor-conditioned media (TCM) *in vitro*. TCM increased the levels of IL-1β via glucose-mediated activation of the inflammasome. Moreover, TCM enhanced the activation of both NF-κB and mTOR pathways in a glucose-dependent manner. In particular, the expression levels of mTORC1 component proteins were dependent on the TCM-induced activation of NF-κB signaling. In addition, TCM affected ASC-ASC interactions through increasing intracellular reactive oxygen species levels. Finally, glucose inhibition by inoculation with 2-deoxy-D-glucose *in vivo* decreased the IL-1β levels in both the blood and tumor region of B16F10-bearing C57BL/6 mice relative to those in PBS-injected tumor-bearing mice. These results suggest that glucose supplied from blood vessels might be important for IL-1β production in tumor-associated macrophages via the integrated signals of the NF-κB and mTOR pathways in the tumor microenvironment.

## Introduction

Tumors are formed by an accumulation of abnormal cells and are uncommon tissues composed of not only tumor cells but also extracellular matrix (ECM), epithelial cells, endothelial cells, adipocytes, fibroblasts and immune cells [1]. It is particularly important to understand the interactions between cancerous cells and macrophages in tumor tissues because cancer cells can alter macrophages via tumor-secreted factors for their survival against host immunosurveillance [2]. For example, lactic acid produced by tumor cells can induce polarization from M0 to M2-like macrophages through inducing the expression of vascular endothelial growth factor (VEGF) and arginase-1 [3]. Macrophage colony-stimulating factor (M-CSF) secreted from MDA-MB231 cells, a human breast cancer cell line, was shown to skew macrophages towards an M2-like phenotype [4]. Furthermore, HMGB1 released from tumor cells can increase the pro-tumoral activities of macrophages via a RAGE-dependent mechanism [5]. In addition, macrophages can be polarized to M2 type through the stimulation of anti-inflammatory cytokines, such as IL-4, IL-13, TGF-β, and IL-10, that are released from the tumor microenvironment [6]. Therefore, tumor secreted factors skew macrophages towards becoming M2-like tumor-associated macrophages (TAMs) that play pro-tumoral roles in tumor development and progression.

Although tumor-associated macrophages have characteristics of the M2-like phenotype, this cell population can also produce pro-inflammatory cytokines, such as IL-1β, depending on the conditions of the tumor microenvironment [7]. For example, tumor secreted factors, including lactate and HMGB1, induced macrophage polarization to M2 type, but these factors also induced IL-1β production in the cells [8, 9]. IL-1β is a general pro-inflammatory cytokine that binds to its receptors and then mediates pyrogenesis and inflammation through the gene expression of various anti-cancer cytokines, prostaglandins, and nitric oxide [10]. However, recent studies have reported that IL-1β in tumor tissues is involved in tumor development and progression [11–14]. Macrophage-derived IL-1β enhanced the growth rate of HCT116 and Hke-3 colorectal carcinoma cell lines via activating Wnt signaling [15]. Compared with control Lewis lung carcinoma (LLC), LLC cells that were transduced with the human IL-1β gene secreted 2-fold the amount of angiogenic VEGF [12]. Likewise, the roles of IL-1β in the tumor microenvironment are controversial. It is also unclear which mechanisms of IL-1β production in TAMs are activated by tumor-secreted factors in the tumor microenvironment. Therefore, it is necessary to clarify the production mechanism of IL-1β in the tumor microenvironment.

Similar to other pro-inflammatory cytokines, IL-1β protein expression is associated with activation of the NF-κB pathway [16]. The degradation of IκBα via its phosphorylation and ubiquitination is required for the nucleus translation of NF-κB [17]. In addition, NF-κB activation has been reported to be dependent upon glucose concentrations in metabolic diseases, such as diabetes [18]. IL-1β gene expression can also be enhanced as a transcription factor of HIF1α that can be induced by activation of the mTOR/S6K pathway [19]. Therefore, IL-1β production could be closely linked to the activation of glucose-mediated mTOR [20]. Moreover, it is a well-known fact that inflammasome activation can produce mature IL-1β in various pathological environments, such as infectious and autoimmune diseases [21]. The inflammasome machinery for IL-1β maturation comprises NLRs (including NLRP1, NLRP6, NLRP12, NLRC5, NLRC4, and NLRP3), ASC and caspase-1 [22]. In particular, NLRP3 inflammasome activation can be mediated by the enhanced intracellular reactive oxygen species (ROS) in macrophages [23]. The NLRP3 inflammasome is fully characterized as an IL-1β-producing agent in various environments [24]. However, the precise mechanisms of IL-1β production and maturation in the tumor microenvironment are largely unknown. To determine the mechanisms of IL-1β production through inflammasome activation in the tumor microenvironment, bone marrow-derived macrophages (BMDMs) exposed to B16F10-conditioned media were investigated.

## Materials and methods

### Mice

C57BL/6 mice (male, wild-type) were obtained from Nara Biotech (Seoul, Republic of Korea), and were used for experiments at 6–8 weeks of age. All mice were housed under pathogen-free conditions at 24–26°C and 40–60% relative humidity with a 12 h light/dark cycle for the duration of the experiments. These mice were also fed a standard diet and water. All mice used in the experiments were sacrificed by cervical dislocation.

### Ethics statement

The animal research was conducted according to the KFDA guidelines for the Care and Use of Laboratory Animals. The experimental design in the present study has been approved and confirmed by the local ethical committee for animal experimentation, KNU (KIACUC; approval number, KW-140711-2). All tumor-bearing mice were monitored for body weight change and for tumor volume measurement every morning until the end of experiments. In our tumor model, the weight of tumor-bearing mice was hardly reduced compared to the weight of non-tumor mice for 14 days. No abnormal signs were also seen in these mice during this period.

### BMDM isolation

L929 mouse fibroblast cells (Korean Cell Line Bank, Seoul, Korea, 10001) were used to differentiate BMDMs from bone marrow precursor cells. Cells were plated and maintained in RPMI supplemented with 10% FBS and 1% antibiotics in a 10-cm culture dish at 37°C and 5% CO_2_ for 5 days. As a source of M-CSF, the supernatant was collected and then filtered through a 0.2-μm filter. The isolation of BMDMs obtained from C57BL/6 mice was carried out as described previously [25]. Briefly, after euthanasia by cervical dislocation, the tibia and femur from each hind leg were obtained. 1-ml syringe with a 26-G needle was used to flush the bones with FBS-free DMEM until the bone cavities appeared to be white. The cell pellet was harvested by centrifugation and suspended in DMEM containing 20% FBS and 30% L929-conditioned media. Then the cells were cultured in a 100-mm petri dish at 5 × 10^6^ cells/dish for 5 days. Polarized BMDMs were used for the tumor microenvironment experiments.

### Preparation of tumor-conditioned media (TCM)

Supernatant obtained from B16F10 cells (Korean Cell Line Bank) was used to mimic the tumor microenvironment [3]. B16F10 cells were seeded and cultured with complete DMEM in a 100-mm cell culture dish at 37°C and 5% CO_2_ until confluency (~90%). The supernatant was collected and then filtered through a 0.2-μm filter. The filtered-supernatant was aliquoted and stored at -20°C until used. We named the B16F10-conditioned media “100% TCM” which was regarded as glucose-depleted because these cells potentially consumed nearly all the glucose in complete DMEM while growing. Two types of TCM mixture (% of TCM) composed of B16F10-conditioned media and DMEM were used; one was “glucose-containing TCM mixture” that consisted of 100% TCM and 25 mM glucose-containing complete DMEM, and the other was a “glucose-depleted TCM mixture” that was composed of 100% TCM and glucose-free DMEM.

### Reagents

For the inhibitor experiments, BMDMs were pre-treated with DMSO (vehicle control) or 50 μM Ac-YVAD-CMK for 1 h (caspase-1 inhibitor; Calbiochem, La Jolla, CA, USA), 1 μM bortezomib for 2 h (IκBα degradation inhibitor; Cell Signaling Technology, Danvers, MA, USA), 10 nM rapamycin for 1 h (mTOR inhibitor; Cell Signaling Technology), 20 μM diphenyleneiodonium chloride for 1 h (DPI; NADPH oxidase inhibitor; Sigma-Aldrich, St. Louis, MO, USA), and 1 mM 2-DG for 1 h (glucose antagonist; Sigma-Aldrich). To determine the effects of intratumoral glucose levels, BMDMs were stimulated with 25% TCM that was mixed with glucose-free DMEM containing 0, 6.25, 12.5, or 25 mM D-glucose (Sigma-Aldrich) for the indicated times.

### Antibodies

The following antibodies were used for western blotting: NLRP3 rabbit mAb (#15101), ASC rabbit mAb (#67824), IL-1β mouse mAb (#12242), phospho-NF-κB p65 rabbit pAb (Ser536; #3033), phospho-IκBα mouse mAb (Ser32/36; #9246), total-IκBα rabbit mAb (#9242), phospho-mTOR rabbit mAb (Ser2448; #2971), total-mTOR rabbit mAb, phospho-p70 S6 Kinase rabbit mAb (Ser371; #9208), raptor rabbit mAb 46, DEPTOR rabbit mAb (#11816), PRAS40 rabbit mAb, anti-mouse IgG (#7076), and anti-rabbit IgG (#7074) were purchased from Cell Signaling Technology Inc. (Beverly, MA, USA). Caspase-1 p10 rabbit pAb (sc-514), total-NF-κB p65 rabbit pAb (sc-7151), total-p70 S6 Kinase mouse mAb (sc-8418), and β-actin mouse mAb (sc-47778). All antibodies were obtained from Santa Cruz Biotechnology Inc. (Santa Cruz, CA, USA).

### Western blotting

BMDMs were harvested, and whole cell lysates were generated using RIPA buffer supplemented with Halt protease and phosphatase inhibitor cocktail (Thermo Scientific Pierce, Rockford, IL, USA) [26]. The cell lysates were quantified by using the Bradford method. Each sample was separated by SDS-PAGE. The membranes were incubated in blocking solution containing 5% skim milk in TBS-T buffer for 1 h and then with specific primary and secondary antibodies for 2 h at room temperature. The membranes were washed three times with TBS-T buffer for 10 min. The membranes were developed using an ECL solution (Advansta, Menlo Park, CA, USA) according to the manufacturer's instructions. Then, images were taken using an AGFA X-ray film (Agfa-Gevaert NV, Mortsel, Belgium).

### Chemical crosslinking assay

ASC oligomerization assays were performed as previously described [27]. Briefly, cells were lysed in buffer A by passing the lysate through a 21-gauge needle 20 times. The cell lysate were centrifuged at 6000 rpm for 10 min. Then, the supernatants were diluted with one volume of CHAPS buffer and centrifuged to pellet the ASC oligomeric complex. Next, the ASC oligomers were cross-linked with 1 mM disuccinimidyl suberate (DSS; Thermo Scientific Pierce Inc., Rockford, IL, USA) for 30 min. The cross-linked pellets and soluble lysates were fractionated on SDS-PAGE and immunoblotted with an anti-ASC antibody.

### siRNA transfection

To silence NF-κB p65, BMDMs were transfected with control siRNA or NF-κB p65siRNA (100 nM; Santa Cruz Biotechnology Inc., Dallas, TX, USA; sc-37007 and sc-29411, respectively) using Lipofectamine RNAiMAX reagent (Invitrogen, Carlsbad, CA, USA) according to the manufacturer's instructions.

### Mouse IL-1β detection by ELISA

Mouse IL-1β was evaluated using a Murine IL-1β Mini ABTS ELISA Development Kit (Peprotech EC Ltd, London, UK) according to the manufacturer's instructions. Briefly, 96-well flexible plates (BD Biosciences, Franklin Lakes, NJ, USA) were coated with 100 μl of the capture antibody and incubated at room temperature overnight. The plates were washed three times with PBS-T, and each well was filled with blocking buffer for 1 h. Then, murine IL-1β standard and supernatants were incubated in the wells for 2.5 h. After washing, each well was treated with 100 μl of the biotin-conjugated detection antibody for 2 h. After washing, avidin-HRP conjugate was incubated for 30 min. Secreted IL-1β was evaluated using 2, 20-azino-bis(3-ethylbenzthiazoline-6-sulphonic acid (ABTS) tablets (Sigma-Aldrich). The plates were read at 490 nm with an ELISA reader (PowerWave XS, BioTek Instruments, Inc., Winooski, VT, USA).

### Confocal microscopy

BMDMs were seeded onto glass coverslips (1 × 10^5^ cells per coverslip; Paul Marienfeld GmbH& Co., KG, Lauda-Könighofen, Germany) in 12 well plates for 24 h [28]. The cells were stimulated with 25% TCM for the indicated times. After incubation, the cells were fixed with 4% paraformaldehyde at room temperature for 15 min. Then, the cells were permeabilized with 0.2% Triton X-100 in PBS for 20 min. After blocking with 1% BSA in PBS for 1 h, specific primary antibodies with 1% BSA and 0.05% Triton X-100 in PBS were incubated for 2 h. In addition, fluorescein isothiocyanate-conjugated BS-I isolectin B4 (Sigma-Aldrich) was used to stain intratumoral blood vessels, and MitoSOX Red (Thermo Fisher Scientific, Waltham, MA, USA) was used to detect mitochondrial ROS; both of these solutions were incubated for 2 h. The secondary antibodies anti-rabbit-FITC and anti-mouse-rhodamine (Jackson ImmunoResearch Laboratories, Inc., West Grove, PA, USA; 111-095-003 and 715-025-150, respectively) with 1% BSA and 0.05% Triton X-100 in PBS were incubated for 2 h. Each well was washed twice with 0.02% Tween 20 and 1% BSA in PBS and then incubated for 5 min with 300 nM 4′, 6-diamidino-2-phenylindole (DAPI; Sigma-Aldrich, St. Louis, MO, USA). The coverslips were mounted, and the cells were observed under a confocal microscope (FV1000; Olympus Corporation, Tokyo, Japan). The results were quantified using a densitometer (ImageJ, version 1.48, National Institutes of Health, Bethesda, MD, USA).

### *In vivo* studies

Briefly, C57BL/6 male mice (5–8 weeks old) were intravenously injected with 5 × 10^5^ B16F10 cells to establish a lung metastasis model [29]. On the next day, the mice were intraperitoneally injected with PBS (vehicle control) or 2-DG (50 mg/kg) in PBS for 5 days. The mice were sacrificed by cervical dislocation on day 14 after tumor cell injection. The lungs and cardiac blood were obtained from these mice for ELISA and western blotting assays. To evaluate effects of 2-DG on tumor progression and intratumoral IL-1β levels, C57BL/6 mice (5–8 weeks old) were subcutaneously injected with 5 × 10^5^ B16F10 cells. PBS or 2-DG (50 mg/kg) in PBS was subcutaneously injected into the tumoral regions on days 7–14, and the tumor tissues were extracted on day 14. Following tumor volume measurements using the Ellipsoid formula (*a × b × c × π × 4/3*), B16F10-derived tumor tissues were frozen in FSC 22 (Surgipath, Richmond, IL, USA). Next, 5-μm cryosections were cut using a Leica CM1100 cryostat (Leica Microsystems GmbH, Wetzlar, Germany) with microtome blades (Leica 818, Leica Microsystems GmbH, Wetzlar, Germany). Then, the tumor tissues were mounted on poly-L-lysine-coated glass slides (Muto Pure Chemicals, Tokyo, Japan). Tumor tissues were stained with anti-IL-1β, IB-4, and DAPI and analyzed by confocal microscopy.

### Whole lung homogenization

Whole lungs obtained from control or tumor-bearing C57BL/6 mice were homogenized as described previously [30]. Briefly, whole lungs were homogenized in 1.5 ml of PBS supplemented with Halt protease and phosphatase inhibitor cocktail (Thermo Scientific Pierce, Rockford, IL, USA). After centrifugation for 20 min at 14,000 rpm and 4°C, the supernatant were collected for ELISA. Each pellet was lysed in RIPA buffer supplemented with protease and phosphatase inhibitor cocktail for western blot assays.

### Statistical analysis

All data are presented as the mean ± SD. Statistical analyses were performed using unpaired t-tests by GraphPad Prism 5 software (GraphPad Software, Inc., La Jolla, CA, USA). P values < 0.05 were considered significant.

## Results

### Tumor-conditioned media induce IL-1β maturation via caspase-1 activation in mouse macrophages in a nutrient-dependent manner

**Fig 1:**
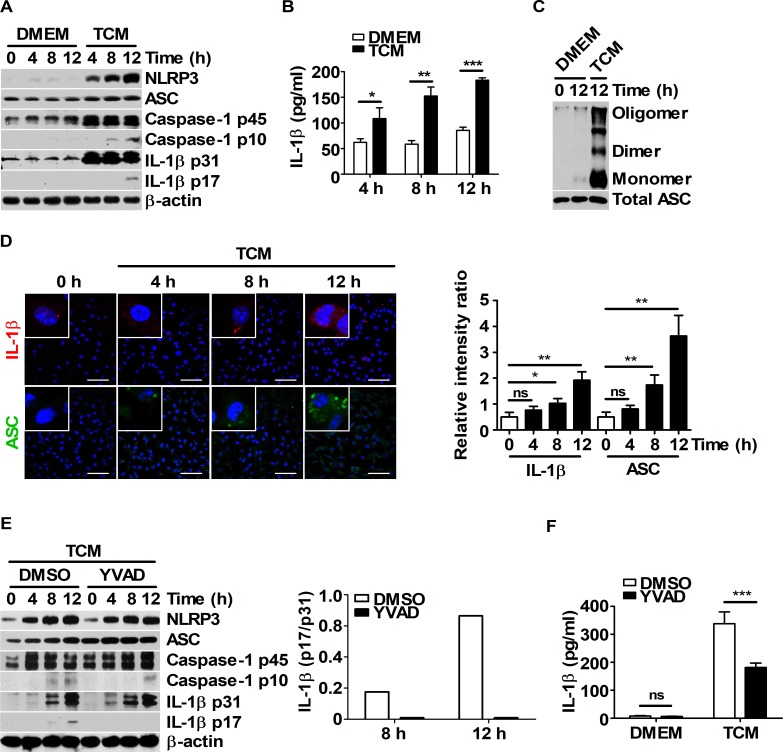
IL-1β maturation is induced in TCM-treated BMDMs via caspase-1 activation. (A-D) BMDMs were incubated with DMEM or 25% TCM for 4, 8, and 12 h. (A) Western blots for NLRP3, ASC, caspase-1, and IL-1β were performed. β-actin was used as the loading control. (B) Secreted IL-1β levels in the supernatants were determined by ELISA. (C) ASC oligomerization was analyzed by DSS chemical crosslinking assay. (D) Intracellular IL-1β (red) and ASC (green) levels were evaluated by confocal microscopy. Scale bars, 50 μm. (E) BMDMs were pre-treated with DMSO or 50 μM YVAD for 1 h before stimulation with DMEM or 25% TCM for 4, 8 and 12 h. Western blots for NLRP3, ASC, caspase-1, and IL-1β were performed. β-actin was used as the control. Bar graph represented the ratio of expression level of IL-1β (p17) versus IL-1β (p31) (right panel). (F) IL-1β secretion levels for (E) were analyzed by ELISA. The bars and error bars represent the mean ± SD; *, P < 0.05; **, P < 0.01; ***, P < 0.001; ns, not significant.

To explore whether IL-1β maturation could be induced by TCM through inflammasome activation in macrophages, BMDMs were stimulated with various concentrations of TCM (25, 50, 75, and 100%; volume : volume), as a source of tumor-secreted factors. The expression levels of NLRP3, ASC, caspase-1, and IL-1β were higher in the 25% TCM-treated BMDM group than in the other groups (Panel A in S1 Fig). Secreted IL-1β levels and ASC-ASC interactions were also increased in the 25% TCM group compared to those in the other groups (Panel B and C in S1 Fig). 25% TCM stimulation in medium with sufficient nutrients resulted in higher levels of mature IL-1β via enhanced inflammasome activation in macrophages (S1 Fig). To elucidate the mechanism of IL-1β maturation in BMDMs stimulated with 25% TCM, BMDMs were treated with DMEM or 25% TCM for the indicated times (Fig 1). Treatment with 25% TCM increased the maturation and secretion of IL-1β (Fig 1A and 1B) and enhanced ASC-ASC interactions in BMDMs (Fig 1C). Increased levels of intracellular IL-1β and ASC speck were also observed by confocal microscopy (Fig 1D). To determine whether caspase-1 activation was associated with the 25% TCM-induced IL-1β maturation, BMDMs were pre-treated with DMSO or YVAD before treatment with 25% TCM. Compared to DMSO, pre-treatment with YVAD remarkably decreased the cleaved form (p17) and secretion levels of IL-1β in BMDMs (Fig 1E and 1F). Taken together, these results suggest that TCM induced IL-1β maturation via caspase-1 activation through the inflammasome platform in BMDMs in a nutrient-dependent manner.

### Glucose supply is needed for the IL-1β maturation via the TCM-induced activation of inflammasome

**Fig 2:**
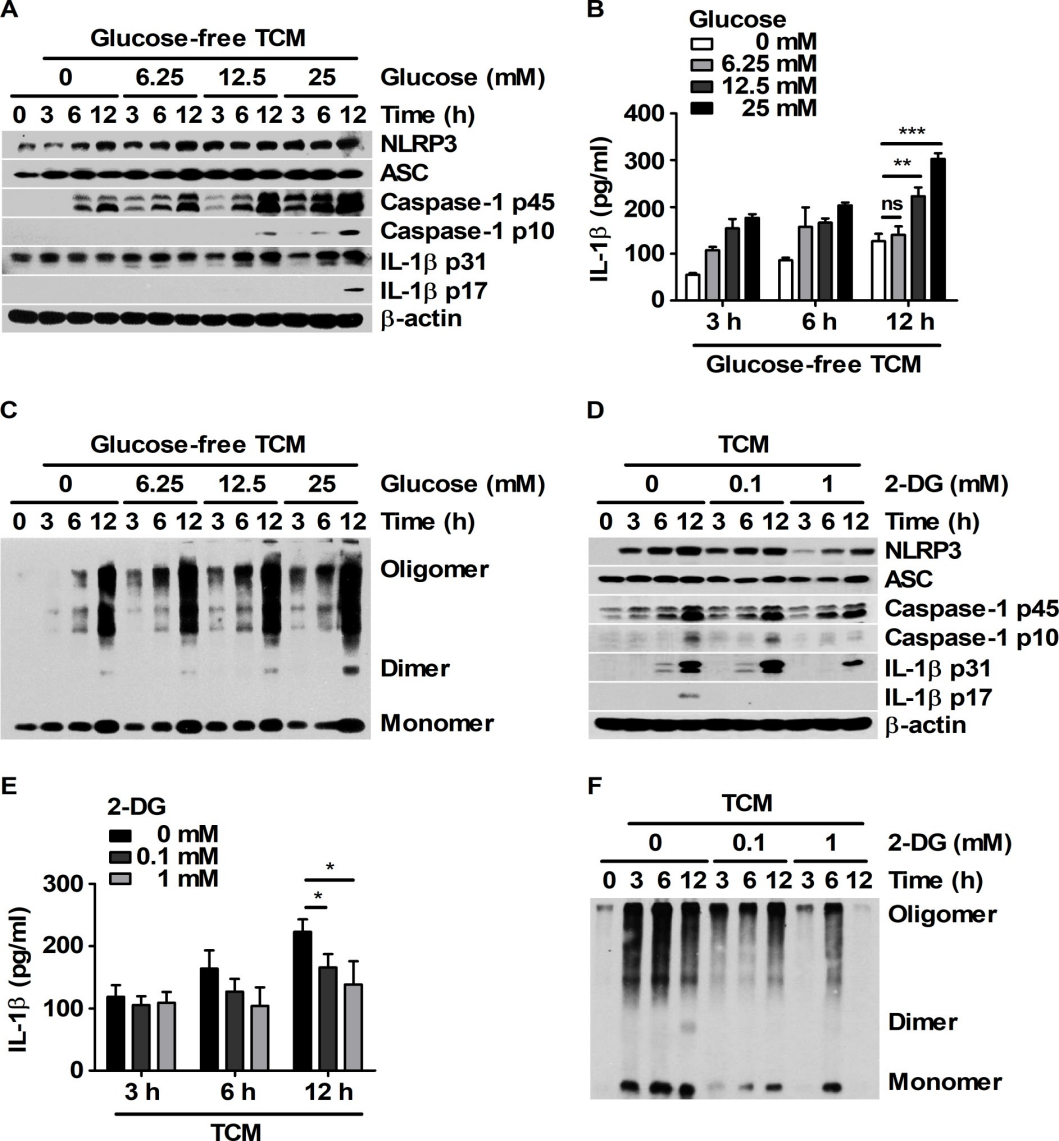
Mature IL-1β was generated by TCM stimulation through glucose-dependent inflammasome activation in BMDMs. (A-C) BMDMs were cultured with 25% TCM mixed with glucose-free media supplemented with 0, 6.25, 12.5 or 25 mM glucose for 3, 6, and 12 h. (A) Western blots for NLRP3, ASC, caspase-1, and IL-1β were performed. β-actin was used as the control. (B) Secreted IL-1β levels in the supernatant were analyzed by ELISA. (C) ASC oligomerization was analyzed by DSS chemical crosslinking assay. (D-F) BMDMs were pre-treated with DMSO or 2-DG (0.1 or 1 mM) for 1 h and then treated with 25% TCM for 3, 6, and 12 h. (D) NLRP3, ASC, caspase-1, and IL-1β levels in the harvested cells were measured by western blot. β-actin was used as the control. (E) IL-1β levels were analyzed by ELISA. (F) ASC oligomerization was analyzed by DSS chemical crosslinking assay. The bars and error bars represent the mean ± SD; *, P < 0.05; **, P < 0.01; ***, P < 0.001; ns, not significant.

Glucose metabolism is required for NLRP3 inflammasome activation in LPS-primed macrophages exposed to ATP [20]. Thus, whether nutrient-dependent IL-1β production is associated with the glucose levels in TCM stimulation was investigated. To determine the effects of glucose in TCM on macrophages, BMDMs were stimulated with 25% TCM that was mixed with glucose-free DMEM containing various concentrations of glucose (0, 6.25, 12.5, and 25 mM). Compared to TCM containing lower concentrations of glucose, TCM containing 25 mM glucose increased the expression levels of NLRP3, caspase-1, and IL-1β in BMDMs (Fig 2A). In addition, the levels of mature and secreted IL-1β were also higher in the 25 mM glucose group than in the other groups at 12 h (Fig 2A and 2B). Notably, glucose-dependent ASC-ASC interactions were observed in BMDMs treated with TCM (Fig 2C). To clarify the role of glucose in inflammasome activation, BMDMs were pre-treated with 2-deoxy-D-glucose (2-DG) before treatment with 25 mM glucose-containing 25% TCM (Fig 2D, 2E and 2F). 2-DG attenuated the expression levels of inflammasome-related proteins and the levels of ASC-ASC assembly (Fig 2D and 2F). Moreover, 2-DG treatment decreased the levels of mature and secreted IL-1β in a dose-dependent manner (Fig 2D and 2E). These results suggest that a sufficient supply of glucose could be required for the maturation and secretion of IL-1β in BMDMs challenged with tumor-secreted factors.

### TCM induce the activation of both NF-κB and mTOR signaling pathways in a glucose-dependent manner

**Fig 3:**
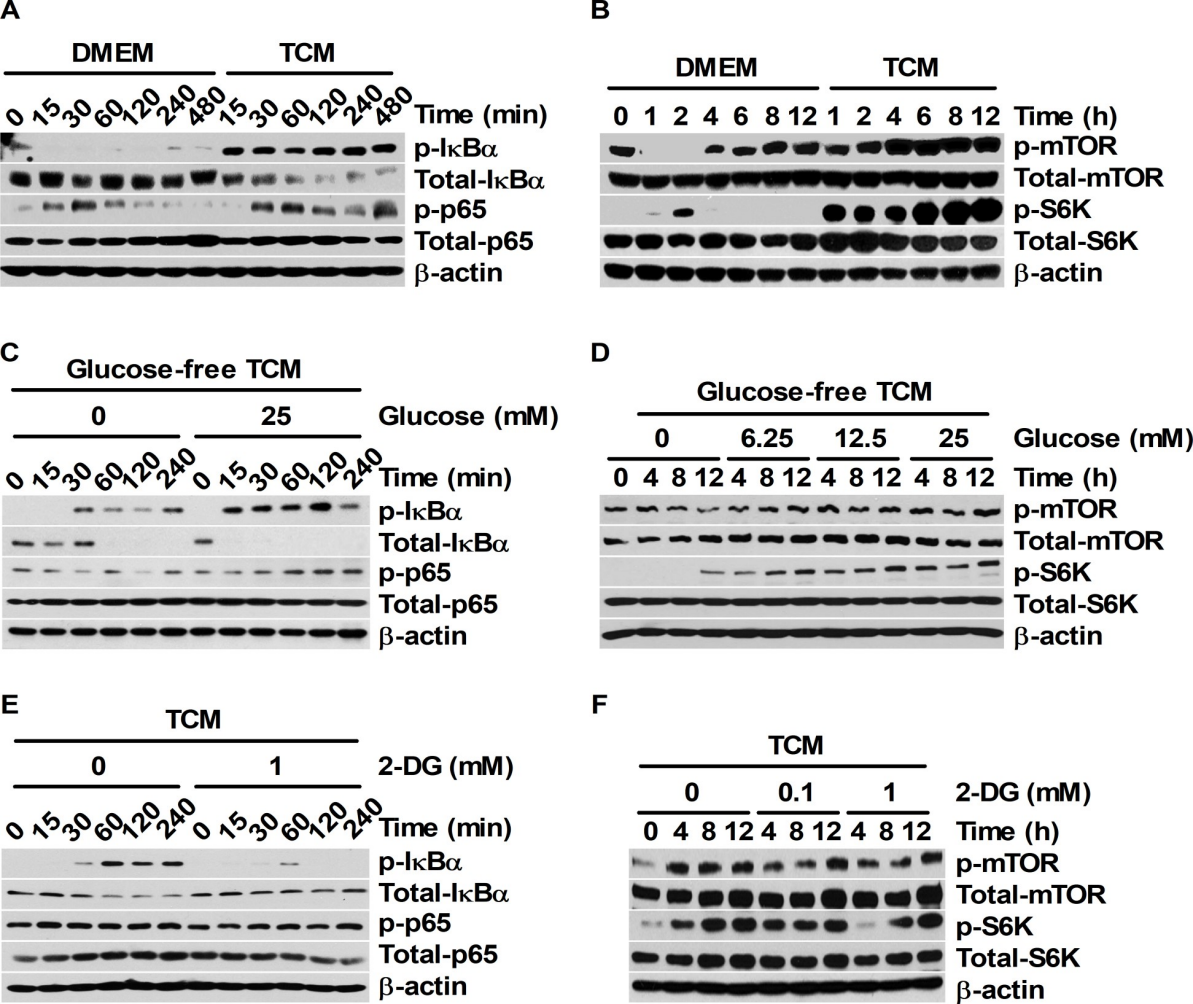
TCM activates both NF-κB and mTOR signaling pathways in a glucose-dependent manner. (A and B) BMDMs were stimulated with 25% TCM for the indicated times. (C and D) BMDMs were stimulated with 25% TCM mixed with glucose-containing DMEM (0, 6.25, 12.5, or 25 mM) for the indicated times. (E and F) BMDMs were pre-treated with DMSO or 2-DG (0.1 or 1 mM) for 1 h and then stimulated with 25% TCM for the indicated times. (A, C, and E) Western blots for phospho-IκBα, IκBα, phospho-NF-κB p65, and NF-κB p65 were performed. (B, D, and F) Phospho-mTOR, mTOR, phospho-p70 S6K, and p70 S6K protein expression levels were analyzed by western blot. (A-F) β-actin was used as the control.

IL-1β protein expression is associated with the activation of NF-κB and mTOR signaling [16, 20]. The activation of both NF-κB and mTOR pathways could also be dependent on glucose concentrations [20, 31]. Thus, the relevance of the glucose-mediated maturation ratio of IL-1β and the activation of NF-κB and mTOR signals was investigated under TCM stimulation. Compared to the DMEM control, 25% TCM enhanced the phosphorylation of IκBα (at Ser32/36) and NF-κB p65 (at Ser536) (Fig 3A). In particular, remarkable levels of IκBα degradation were detected in 25% TCM-treated BMDMs (Fig 3A). Notably, the phosphorylation of mTOR at Ser2448 and p70 S6K at Ser371 was strongly induced by 25% TCM treatment in BMDMs (Fig 3B). To determine whether 25% TCM-induced NF-κB and mTOR signaling pathway activation could be affected by the TCM nutrient content, NF-κB and mTOR expression and phosphorylation levels were examined in BMDMs stimulated with various concentrations of TCM (25, 50, 75, and 100%) (S2 Fig). Although 50%, 75%, and 100% TCM also induced the phosphorylation of NF-κB and mTOR signal proteins, 25% TCM significantly enhanced the phosphorylation levels of IκBα, NF-κB p65, mTOR, and p70 S6K (Panel A and B in S2 Fig). To clarify the correlation between the nutrient-dependent phosphorylation of these signal proteins and the glucose levels in TCM, NF-κB and mTOR expression and phosphorylation levels were analyzed in BMDMs stimulated with 25% TCM that was mixed with glucose-free DMEM containing various concentrations of glucose (0, 6.25, 12.5, and 25 mM). Compared to 25% TCM supplemented with glucose-free media, 25% TCM containing 25 mM glucose remarkably increased the phosphorylation levels of NF-κB and IκBα and the degradation level of IκBα (Fig 3C). In contrast, pre-treatment with 2-DG attenuated the phosphorylation levels of NF-κB and IκBα and the degradation level of IκBα in a dose-dependent manner (Fig 3E). Likewise, 25% TCM containing 25 mM glucose enhanced the phosphorylation of mTOR and p70 S6K, but pre-treatment with 2-DG reduced the phosphorylation levels of these proteins (Fig 3D and 3F). Overall, these results suggest that the signal activation of both NF-κB and mTOR that was induced by tumor-secreted factors could be closely associated with glucose concentration in TCM.

### The activation of both NF-κB and mTOR signaling pathways is involved in the TCM-induced maturation and secretion of IL-1β

**Fig 4:**
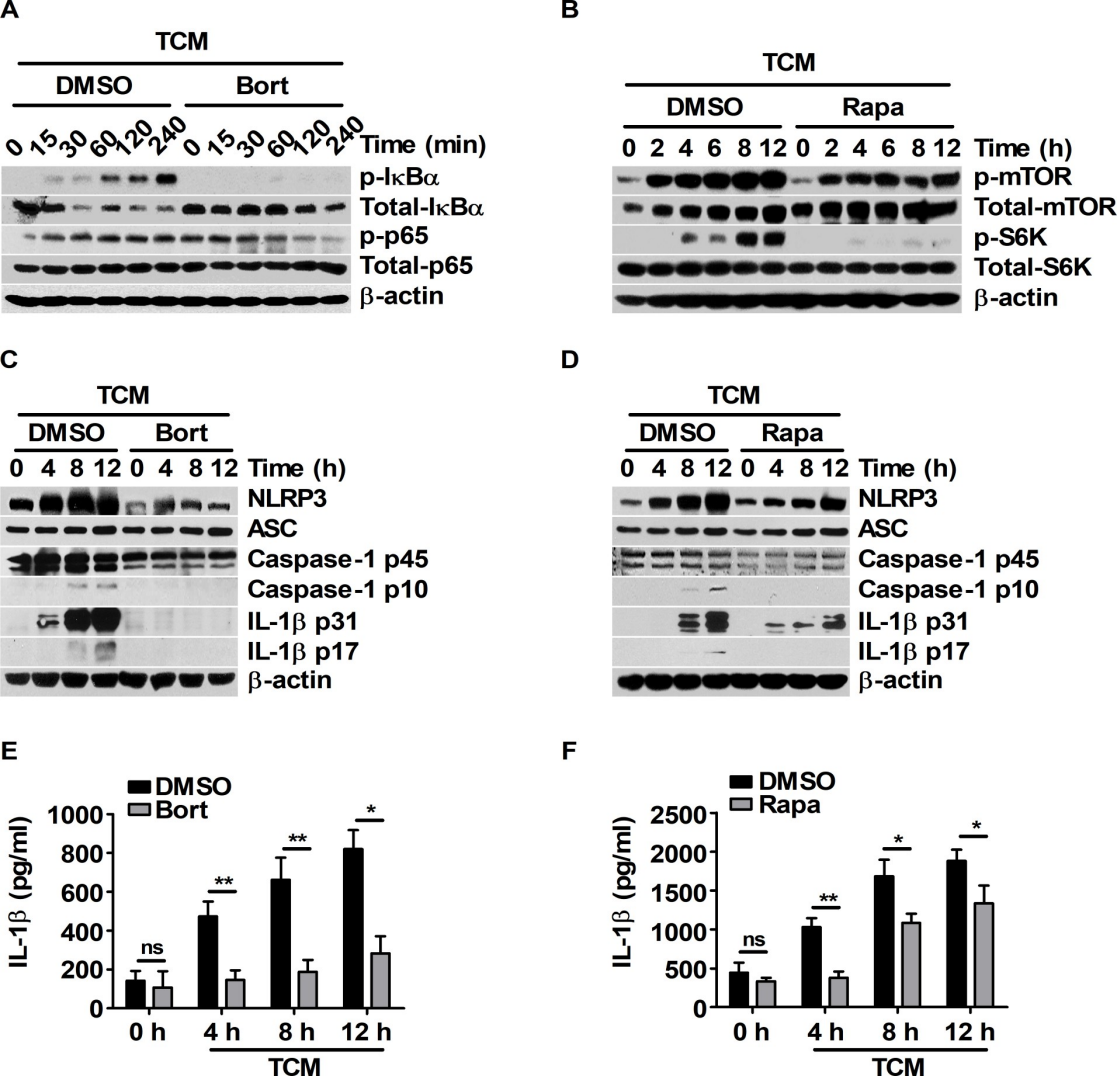
Pharmacologic inhibition of the NF-κB and mTOR pathways suppresses IL-1β maturation in TCM-stimulated BMDMs. (A, C, and E) BMDMs were pre-treated with DMSO or 1 μM bortezomib for 2 h and then stimulated with 25% TCM for the indicated times. (B, D, and F) BMDMs were pre-treated with DMSO or 10 nM rapamycin for 1 h before the cells were treated with 25% TCM for the indicated times. (A) Phospho-IκBα, IκBα, phospho-NF-κB p65, and NF-κB p65 were evaluated by western blot. (B) Phospho-mTOR, mTOR, phospho-p70 S6K, and p70 S6K were analyzed by western blot. (C, D) Cellular expression levels of NLRP3, ASC, caspase-1, and IL-1β were determined by western blot. (E and F) ELISAs were performed to measure the secreted IL-1β levels in (C) and (D). The bars and error bars represent the mean ± SD; *, P < 0.05; **, P < 0.01; ***, P < 0.001; ns, not significant.

To determine the relevance of IL-1β production and signal activation of NF-κB/mTOR upon treatment with 25 mM glucose-containing TCM, the expression levels of inflammasome machinery and the maturation of IL-1β were investigated in BMDMs pre-treated with bortezomib or rapamycin before TCM treatment. Bortezomib and rapamycin effectively inhibited the NF-κB and mTOR pathways, respectively (Fig 4A and 4B). Bortezomib suppressed the expression levels of NLRP3 and caspase-1 p45 (Fig 4C). In particular, IL-1β maturation and secretion were noticeably abolished by bortezomib (Fig 4C and 4E). Compared to DMSO pre-treatment, rapamycin pre-treatment attenuated the expression levels of NLRP3, caspase-1 p45, and IL-1β p31 (Fig 4D). Rapamycin also decreased the maturation and secretion of IL-1β (Fig 4D and 4F). These results suggest that TCM induced IL-1β maturation and secretion through the signal activation of both NF-κB and mTOR pathways. To elucidate the signal interaction between the NF-κB and mTOR pathways under TCM stimulation, the signal pathways of NF-κB and mTOR were analyzed in BMDMs pre-treated with bortezomib or rapamycin before TCM treatment. Rapamycin did not affect the phosphorylation levels of NF-κB (Panel A in S3 Fig), but bortezomib reduced the phosphorylation levels of mTOR and p70 S6K (Panel B in S3 Fig). Bortezomib also decreased the TCM-induced expression of mTOR complex 1-related proteins, such as raptor, DEPTOR, and PRAS40 (Panel C in S3 Fig). To determine whether the side effects of bortezomib induced the expression of mTOR complex 1-related proteins, p65 siRNA was transfected into BMDMs before TCM treatment. Optimized NF-κB p65 knockdown was verified by western blot analysis after transfection (Panel D in S3 Fig). Compared to control siRNA transfection, p65 siRNA transfection attenuated the expression of mTOR complex 1-related proteins in TCM-stimulated BMDMs (Panel E in S3 Fig); these results were similar to the effects of bortezomib. Taken together, these results suggest that TCM could induce the activation of both NF-κB and mTOR signals. In particular, TCM-induced activation of the mTOR pathway could be regulated by the expression of mTOR complex 1-related proteins that are induced by NF-κB activation.

### IL-1β maturation and secretion require intracellular ROS generation under TCM stimulation

**Fig 5:**
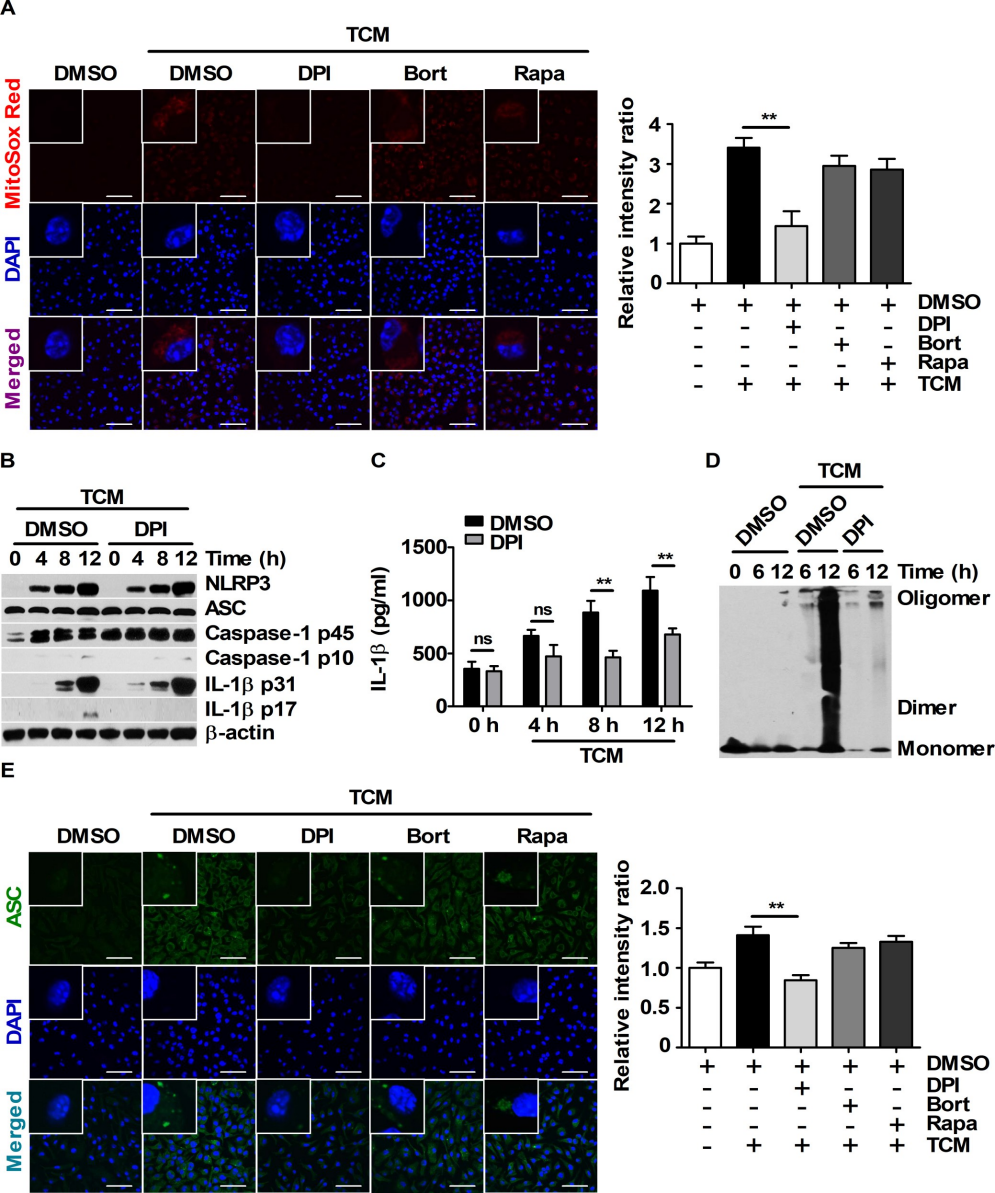
Enhanced ASC-ASC interactions are related to intracellular ROS levels in TCM-stimulated BMDMs. (A and E) BMDMs were pre-treated with DMSO, 20 μM DPI, 5 μM bortezomib or 10 nM rapamycin for 1 h. Then, the cells were stimulated with 25% TCM. (A) Mitochondrial ROS localization in BMDMs stimulated with DMEM or 25% TCM for 6 h was visualized by MitoSOX Red staining. Nuclei (blue) were stained with DAPI. Scale bars, 50 μm. (B-D) BMDMs were pre-treated with DMSO or 20 μM DPI for 1 h and then stimulated with 25% TCM for the indicated times. (B) Protein levels of NLRP3, ASC, caspase-1, and IL-1β were measured by western blot. (C) The supernatant obtained in (B) were evaluated by ELISA. (D) ASC oligomerization was analyzed by DSS chemical crosslinking assay. (E) ASC specks in BMDMs stimulated with 25% TCM were visualized by immunostaining with an anti-ASC antibody (green). Nuclei were stained with DAPI (blue). Scale bars, 50 μm. The bars and error bars represent the mean ± SD; *, P < 0.05; **, P < 0.01; ***, P < 0.001; ns, not significant.

Intracellular ROS are essential for NLRP3 inflammasome activation [23]. To investigate whether intracellular ROS could be involved in TCM-induced inflammasome the activation, ROS levels were evaluated by MitoSOX Red staining in BMDMs pre-treated with diphenyleneiodonium chloride (DPI) before TCM treatment. DPI effectively reduced TCM-induced mitochondrial ROS in BMDMs, whereas bortezomib and rapamycin did not affect the mitochondrial ROS levels (Fig 5A). Interestingly, DPI pre-treatment attenuated IL-1β maturation and secretion (Fig 5B and 5C), but the expression levels of NLRP3, ASC, caspase-1 p45, and IL-1β p31 were not affected by DPI (Fig 5B). To determine the cause of this phenomenon, NF-κB and mTOR signal activation was investigated in BMDMs pre-treated with DPI before TCM treatment. DPI did not affect NF-κB or mTOR signaling in BMDMs stimulated with TCM (Panel F and G in S3 Fig). Because NLRP3 inflammasome assembly is associated with increased mitochondrial ROS levels [32, 33], the role of ASC-ASC interactions in DPI pre-treatment was examined. TCM-induced ASC-ASC interactions were abolished by DPI (Fig 5D). DPI also attenuated the intracellular ASC speck levels (Fig 5E). These results imply that ASC assembly induced by enhanced intracellular ROS levels is required for the maturation and secretion of IL-1β in TCM-stimulated BMDMs.

### Glucose inhibition in tumor-bearing mice through 2-DG injection decreases IL-1β levels *in vivo*

**Fig 6:**
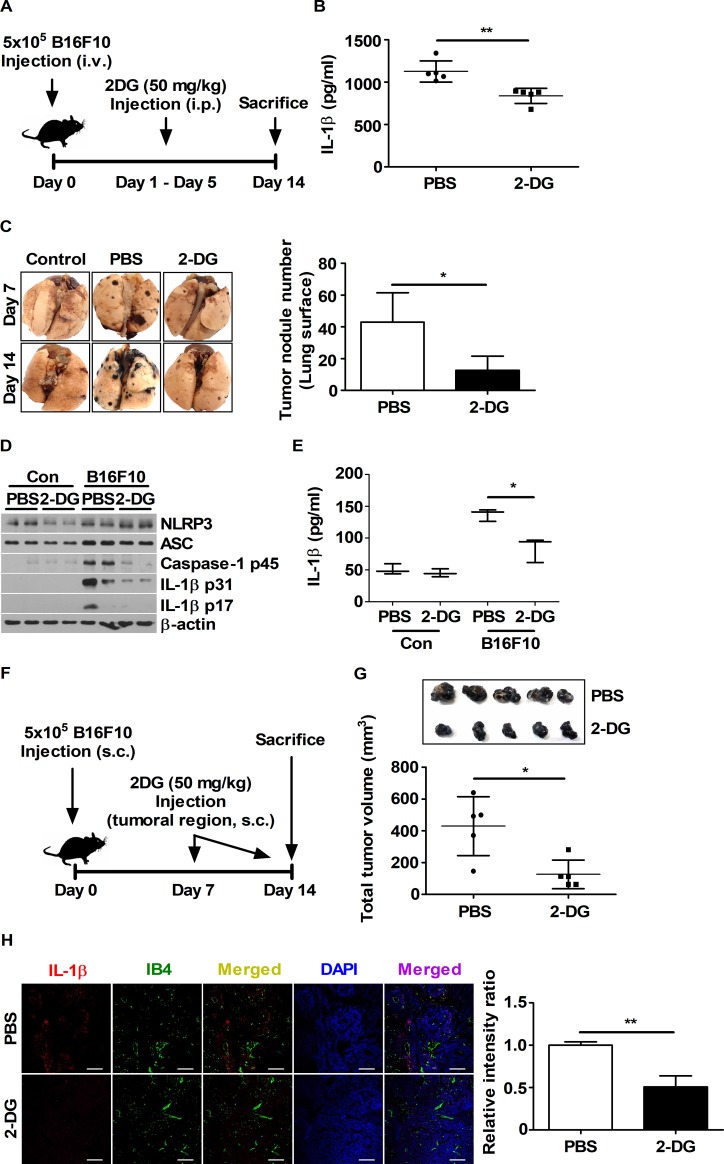
Glucose inhibition decreases IL-1β levels and tumor progression in the B16F10 mouse model. (A and F) Schematic representation of the *in vivo* experimental set up. (B) Plasma IL-1β levels obtained from (A) were evaluated by ELISA at day 7. (C) Images of isolated lungs obtained from (A) at day 7 and day 14 are presented. The number of tumor nodules was scored for the lung surfaces obtained from (A) at day 14. (D) NLRP3, ASC, caspase-1, and IL-1β protein expression levels in homogenized lung were determined by western blot. (E) IL-1β levels in homogenized whole lung were analyzed by ELISA. (G) The volumes of tumor tissues obtained in (F) were measured using calipers. (H) Intratumoral levels of IL-1β (red) and IB4 (green) were evaluated by confocal microscopy. Nuclei (blue) were stained with DAPI. Scale bars, 100 μm. The bars and error bars represent the mean ± SD; *, P < 0.05; **, P < 0.01; ***, P < 0.001; ns, not significant.

To determine whether IL-1β production can be mediated by glucose levels in tumor-bearing mice, C57BL/6 mice injected with B16F10 cells were treated with saline or 50 mg/kg 2-DG for 5 days beginning 1 day after tumor inoculation (Fig 6A). In a lung metastasis model, 2-DG decreased the IL-1β levels in the blood of tumor-bearing mice (Fig 6B). 2-DG inoculation also attenuated the increased expression levels of NLRP3, caspase-1 p45, and IL-1β p31 in tumor-metastasized lungs (Fig 6D). In particular, IL-1β levels were remarkably decreased in homogenized lung tissues from B16F10-bearing mice injected with 2-DG (Fig 6E). In a subcutaneous injection model used to study the tumor microenvironment (Fig 6F), decreased intratumoral levels of IL-1β were observed by confocal microscopy in tumor-bearing mice injected with 2-DG (Fig 6H). IB4 was used as vessel control (Fig 6H). Interestingly, 2-DG also decreased tumor volumes and metastasis (Fig 6C and 6G). Taken together, glucose supply could be associated with IL-1β production in tumor-bearing mice and could affect tumor development and metastasis.

## Discussion

Macrophages that are affected by cancer-secreted factors can facilitate cancer development and progression through differentiation into M2-like TAMs [34]. Like M2 macrophages, TAMs interfere with the entry of anti-cancer immune cells into tumor tissues and create a favorable environment for cancer cells by secreting anti-inflammatory cytokines [1]. However, TAMs can also secrete pro-inflammatory cytokines, such as IL-1β, depending on the conditions in the tumor tissues [35]. In recent studies, positive effects of IL-1β produced by macrophages on tumorigenesis and angiogenesis have been reported [36, 37], but the mechanism of IL-1β production is unknown in the tumor microenvironment. Thus, to elucidate the mechanism of IL-1β production in macrophages stimulated by tumor-secreted factors, BMDMs were treated with B16F10-conditioned media *in vitro*. Higher concentrations of TCM were expected to result in higher IL-1β production in BMDMs because higher TCM concentration means that there are many tumor-secreted factors in the media. However, higher levels of inflammasome-related proteins, ASC-ASC interactions, and IL-1β maturation were induced in BMDMs stimulated with TCM supplemented with nutrient-rich complete media. This phenomenon is supported by a previous study indicating that IL-1β production by the NLRP3 inflammasome was mediated by the nutrient-dependent activation of NF-κB signaling in phorbol-12-myristate-13-acetate (PMA)-treated THP-1 [18]. This led us to question which nutrients in TCM could induce IL-1β maturation via inflammasome activation in macrophages. A recent study has shown that IL-1β production through inflammasome activation could be dependent on glucose concentrations [20]. Thus, to determine whether nutrient-dependent IL-1β maturation could be associated with TCM glucose concentrations, BMDMs were stimulated with TCM containing various concentrations of glucose (0, 6.25, 12.5, and 25 mM). TCM-induced IL-1β maturation could be induced in BMDMs in a glucose-dependent manner. These results suggest that glucose supply might be required for IL-1β maturation via inflammasome activation in TAMs in the tumor microenvironment.

The production of many pro-inflammatory cytokines has been reported to be associated with the activation of transcription factors, such as NF-κB and AP-1 [38]. IL-1β enhancer is activated by NF-κB, AP-1 (Fos/Jun), C/EBPβ, PU.1, IRFs, and STAT [39]. Moreover, NF-κB activity can be modulated in a glucose-dependent manner [40, 41]. In addition to these transcription factors, HIF1α is also an important transcription factor for IL-1β production because it binds to the dimeric pyruvate kinase M2 (PKM2) [42]. It has been reported that enhanced mTOR-S6K pathway activation is involved in HIF1α translation [43] and mTORC1 regulates NLRP3 inflammasome activation via hexokinase 1-dependent glycolysis [20]. All of this evidence supports our observations that IL-1β maturation in TCM-stimulated BMDMs occurs through the glucose-mediated activation of NF-κB and mTOR signaling. Moreover, since both NF-κB and mTOR signals were activated by TCM stimulation, it was investigated which signal was the main contributor to IL-1β production under TCM stimulation. Rapamycin did not affect the signal activation of NF-κB, but bortezomib inhibited the expressions of raptor, DEPTOR, and PRAS40 in BMDMs stimulated with TCM. The same results were obtained when BMDMs were transfected with p65 siRNA instead of the pre-treatment of bortezomib. This phenomenon suggests that TCM-induced IL-1β production could require not only the signal activation of NF-κB pathway but also the signal activation of mTOR via NF-κB-induced the elevated expressions of raptor, DEPTOR, and PRAS40. Our results provide evidence that NF-κB modulates mTOR activation by controlling the expression of its components.

ROS are a stress indicator that can be induced by various intrinsic or extrinsic factors in the tumor microenvironment [44]. It was found that TCM increased the intracellular ROS levels in BMDMs. IL-1β maturation requires not only pro-IL-1β production via its gene transcription but also intracellular ROS accumulation for inflammasome assembly to activate caspase-1. It was observed that DPI decreased TCM-induced IL-1β maturation without changes in the expression of NLRP3, ASC, caspase-1, or pro-IL-1β. These findings are consistent with a previous study of IL-1β maturation in THP-1 cells pre-treated with DPI before treatment with LPS and ATP. Therefore, intracellular ROS accumulation might be essential for inflammasome activation in TAMs in the tumor microenvironment.

Glucose could be an important factor required for IL-1β maturation via inflammasome activation. To determine whether glucose is required for IL-1β production *in vivo*, C57BL/6 mice were injected with 2-DG after B16F10 tumor transplantation. IL-1β levels were increased in tumor-bearing mice, and the inhibition of glucose attenuated IL-1β levels in tumor-bearing mice. These data indicate that IL-1β might be produced via glucose-mediated activation of the inflammasome during tumor progression. Interestingly, 2-DG injection decreased the tumor volumes and reduced the migration ratio of B16F10 into lung tissue. In a previous study, it was reported that 2-DG injection decreased the tumor weights and volumes of PC3 xenografts [45]. These effects of 2-DG on tumor progression are in concordance with our results. Although there is extensive evidence in the literature demonstrating that IL-1β promotes tumor invasiveness and angiogenesis *in vivo* [46], direct evidence for the relation between IL-1β levels and tumor progression have not been found. Therefore, additional investigation is needed for clarifying the pro-tumoral roles of IL-1β.

In conclusion, it was demonstrated that tumor-secreted factors could induce IL-1β maturation via the glucose-mediated signal activation of both NF-κB and mTOR in macrophages in the tumor microenvironment. Furthermore, IL-1β levels were linked to the glucose supply in tumor-bearing mice. Our results provide new insight into the potential pro-tumoral role of IL-1β production in TAMs via NLRP3 inflammasome activation.

## Supporting information

S1 Fig IL-1β expression and its maturation process are induced in BMDMs in a nutrient-dependent manner. (A) BMDMs were exposed to various concentrations of TCM (25, 50, 75, and 100%). Western blots for NLRP3, ASC, caspase-1 and IL-1β were performed. β-actin was used as the standard. (B) Supernatants were evaluated using ELISA. (C) ASC oligomerization was analyzed by DSS chemical crosslinking assay. The bars and error bars represent the mean ± SD; *, P < 0.05; **, P < 0.01; ***, P < 0.001; ns, not significant.
(TIF)

S2 Fig Enhanced signal cascades of the NF-κB and mTOR pathways are related to the TCM nutrient concentration. (A and B) BMDMs were exposed to various concentrations of TCM (25, 50, 75, and 100%). (A) Phospho-IκBα, IκBα, phospho-NF-κB p65, and NF-κB p65 levels were measured by western blot. (B) Western blots for phospho-mTOR, mTOR, phospho-p70 S6K, and p70 S6K were performed. (A and B) β-actin was used as the loading control.
(TIF)

S3 Fig Inhibiting mTOR signaling does not affect NF-κB signaling, but suppressing the NF-κB pathway attenuates the expression of mTOR components. BMDMs were pre-treated with DMSO, 10 nM rapamycin (A), 1 μM bortezomib (B and C), and 20 μM DPI (F and G) for 1 h before stimulation with 25% TCM. (D and E) BMDMs were transfected with control siRNA-A and NF-κB p65 siRNA (100 nM) for 24 h. (A, D, and F) Western blots were performed using anti-phospho-IκBα, anti-IκBα, and anti-NF-κB p65 antibodies. (B and G) Western blots for phospho-mTOR, mTOR, phospho-p70 S6K and p70 S6K were performed. (C and E) Protein expression levels of raptor, DEPTOR, and PRAS40 were measured by western blot. β-actin was used as the loading control for all blots.
(TIF)
