# Supplementary figures and images for: Tumor-secreted factors induce IL-1β maturation via the glucose-mediated synergistic axis of mTOR and NF-κB pathways in mouse macrophages
Source: PLoS One. 2018 Dec 26;13(12):e0209653. doi: 10.1371/journal.pone.0209653 (PMC6306269; doi:10.1371/journal.pone.0209653)

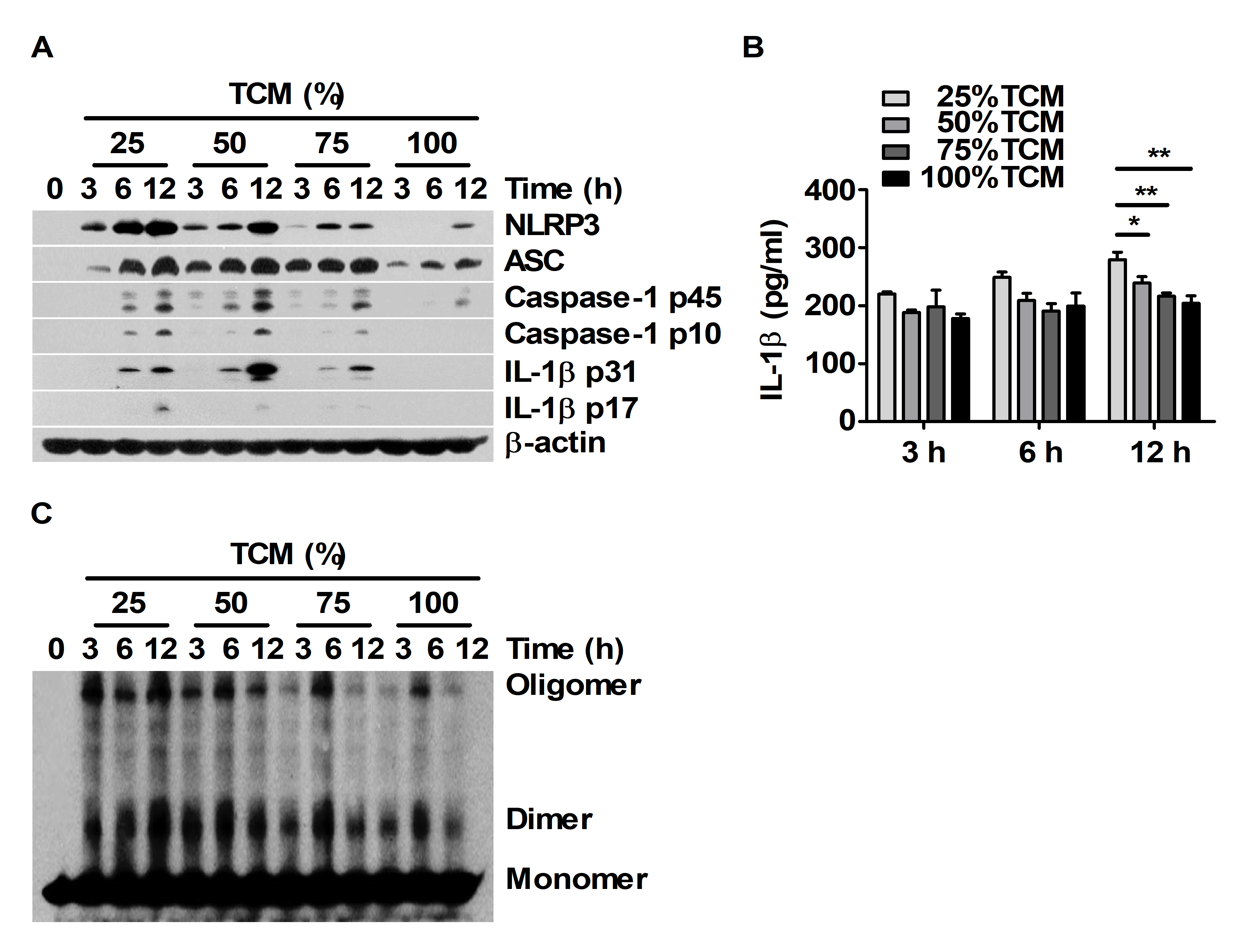

Supplement: S1 Fig — (A) BMDMs were exposed to various concentrations of TCM (25, 50, 75, and 100%). Western blots for NLRP3, ASC, caspase-1 and IL-1β were performed. β-actin was used as the standard. (B) Supernatants were evaluated using ELISA. (C) ASC oligomerization was analyzed by DSS chemical crosslinking assay. The bars and error bars represent the mean ± SD; *, P < 0.05; **, P < 0.01; ***, P < 0.001; ns, not significant. (TIF) [file pone.0209653.s001.tif]

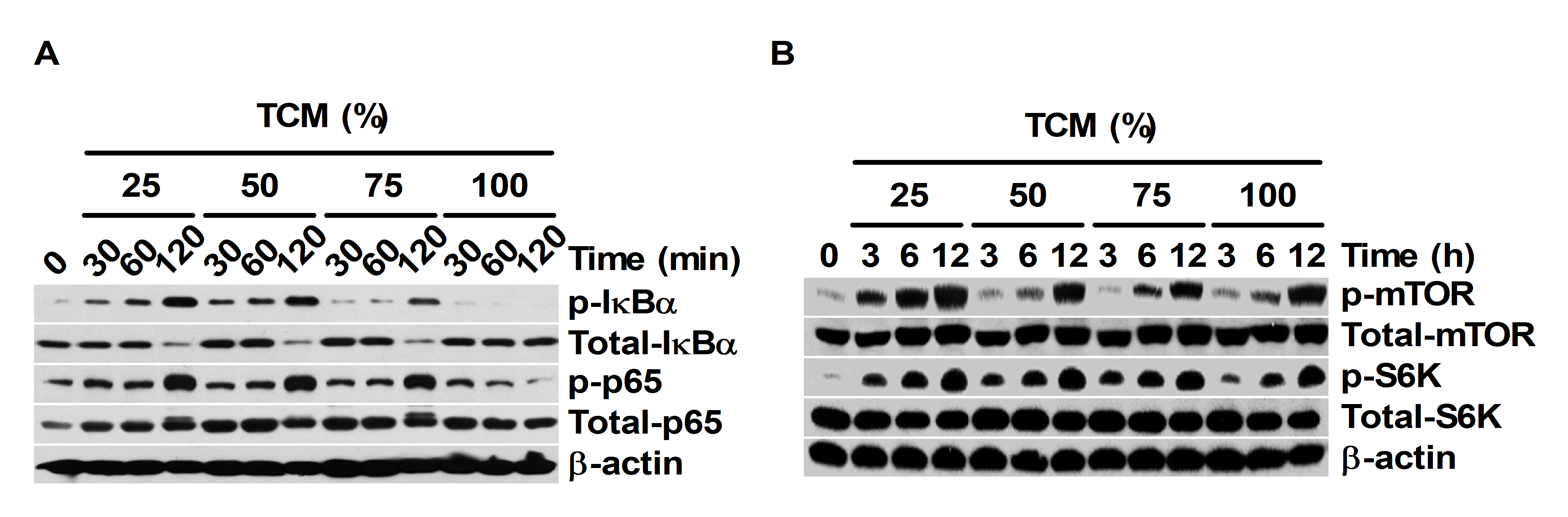

Supplement: S2 Fig — (A and B) BMDMs were exposed to various concentrations of TCM (25, 50, 75, and 100%). (A) Phospho-IκBα, IκBα, phospho-NF-κB p65, and NF-κB p65 levels were measured by western blot. (B) Western blots for phospho-mTOR, mTOR, phospho-p70 S6K, and p70 S6K were performed. (A and B) β-actin was used as the loading control. (TIF) [file pone.0209653.s002.tif]

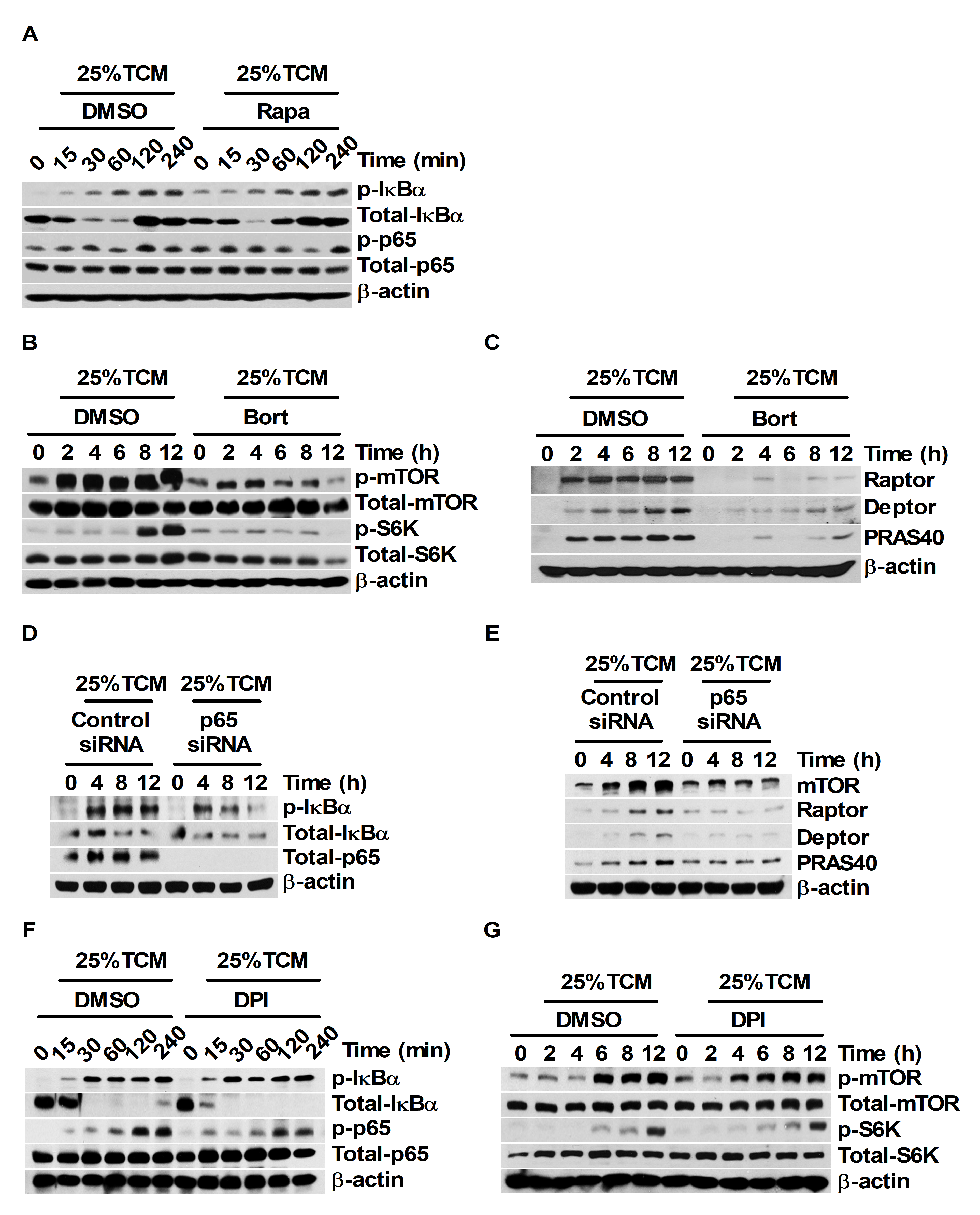

Supplement: S3 Fig — BMDMs were pre-treated with DMSO, 10 nM rapamycin (A), 1 μM bortezomib (B and C), and 20 μM DPI (F and G) for 1 h before stimulation with 25% TCM. (D and E) BMDMs were transfected with control siRNA-A and NF-κB p65 siRNA (100 nM) for 24 h. (A, D, and F) Western blots were performed using anti-phospho-IκBα, anti-IκBα, and anti-NF-κB p65 antibodies. (B and G) Western blots for phospho-mTOR, mTOR, phospho-p70 S6K and p70 S6K were performed. (C and E) Protein expression levels of raptor, DEPTOR, and PRAS40 were measured by western blot. β-actin was used as the loading control for all blots. (TIF) [file pone.0209653.s003.tif]
